# SPring-8 BL41XU, a high-flux macromolecular crystallography beamline

**DOI:** 10.1107/S0909049513022176

**Published:** 2013-10-02

**Authors:** Kazuya Hasegawa, Nobutaka Shimizu, Hideo Okumura, Nobuhiro Mizuno, Seiki Baba, Kunio Hirata, Tomoyuki Takeuchi, Hiroshi Yamazaki, Yasunori Senba, Haruhiko Ohashi, Masaki Yamamoto, Takashi Kumasaka

**Affiliations:** a SPring-8/JASRI, 1-1-1 Kouto, Sayo, Hyogo 679-5198, Japan; b KEK-PF, 1-1 Oho, Tsukuba, Ibaraki 305-0801, Japan; c RIKEN SPring-8 Center, 1-1-1 Kouto, Sayo, Hyogo 679-5148, Japan

**Keywords:** macromolecular crystallography, micro-crystallography, high-flux beam, high-energy beam, SPring-8

## Abstract

SPring-8 BL41XU is a high-flux macromolecular crystallography beamline using an in-vacuum undulator as a light source. The X-rays are monochromated by a liquid-nitrogen-cooling Si double-crystal monochromator, and focused by Kirkpatrick–Baez mirror optics. The focused beam size at the sample is 80 µm (H) × 22 µm (V) with a photon flux of 1.1 × 10^13^ photons s^−1^. A pinhole aperture is used to collimate the beam in the range 10–50 µm. This high-flux beam with variable size provides opportunities not only for micro-crystallography but also for data collection effectively making use of crystal volume. The beamline also provides high-energy X-rays covering 20.6–35.4 keV which allows ultra-high-resolution data to be obtained and anomalous diffraction using the *K*-edge of Xe and I. Upgrade of BL41XU for more rapid and accurate data collection is proceeding. Here, details of BL41XU are given and an outline of the upgrade project is documented.

## Abstract 2

SPring-8 BL41XU provides a high-flux X-ray beam of size 10–50 µm, and enables high-quality diffraction data to be obtained from various types of protein crystals. Details of this beamline and an upgrade project are described.

## 1. Introduction

Synchrotron macromolecular crystallography (MX) beamlines are inevitable tools for the structural analysis of biological macromolecules nowadays. At SPring-8, there are seven MX beamlines which have various specifications for tackling various targets of structural biology (Kawano *et al.*, 2010 ▶). Among them, the four bending-magnet beamlines provide stable beam for high-throughput data collection from well diffracting crystals using automatic data collection systems (Ueno *et al.*, 2004 ▶). The three others are undulator beamlines dedicated to data collection for more challenging targets; BL32XU is a micro-focus beamline whose minimum beam size of 1 µm with a photon flux of 6 × 10^10^ photons s^−1^ achieves data collection from micrometer-sized crystals (Hirata *et al.*, 2013 ▶). The lower divergence beam at BL44XU is suitable for data collection from large-unit-cell crystals (Yoshimura *et al.*, 2007 ▶). BL41XU is a high-flux beamline with an available beam size of 10 µm to 50 µm with photon flux of 6 × 10^11^ to 4 × 10^12^ photons s^−1^. The usable beam size is suitable for data collection from the majority of crystals brought to the synchrotron beamline, and enables both data collection from small crystals down to 10 µm and high-resolution data collection making use of crystal volume. BL41XU is the only public MX undulator beamline at SPring-8, thus it is opened for industrial users, foreign researchers, as well as domestic academic users. Here, we report details of BL41XU and give a summary of the upgrading project.

## 2. Beamline layout, X-ray source and beamline optics

### 2.1. Beamline layout

**Figure 1:**
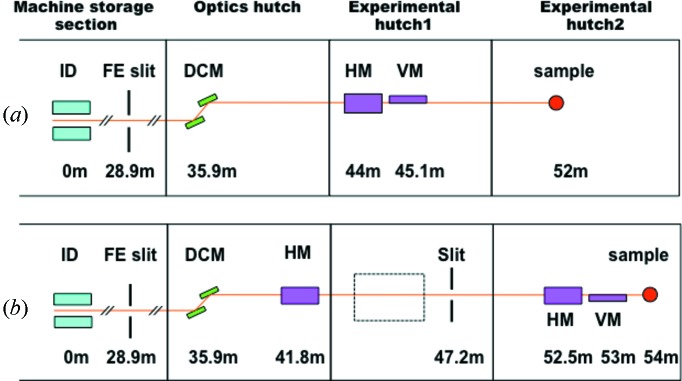
(*a*) Schematic of the optics layout of BL41XU showing distances from the light source. (*b*) Design of the new optics layout for the upgrade project. The slit in the experimental hutch 1 (EH1) is a high-precision slit which defines the virtual source size. The dotted-line rectangle in EH1 shows where the second diffractometer will be installed for the high-energy mode. ID, insertion device; FE slit, front-end slit; DCM, double-crystal monochromator; HM, horizontal-focusing mirror; VM, vertical-focusing mirror.

Fig. 1(*a*) ▶ shows a schematic of the optics layout of BL41XU. BL41XU has three hutches: one optics hutch (OH) and two experimental hutches (EH1 and EH2). The monochromator and focusing mirrors are installed in OH1 and EH1, respectively, and the diffractometer is installed in EH2.

### 2.2. X-ray source and beamline optics

The light source of BL41XU is the SPring-8 standard in-vacuum undulator which covers 5.2–18.5 keV in the first harmonics and 15.5–51 keV in the third harmonics (Kitamura, 1998 ▶). The X-rays are monochromated by the Si(111) double-crystal monochromator (DCM) equipped with a liquid-nitrogen cooling system.

The focusing mirror adopts a Kirkpatrick–Baez (KB) configuration, which consist of a 700 mm horizontal focusing mirror (HFM) and 400 mm vertical focusing mirror (VFM). Both mirrors are coated with rhodium on the flat silicon substrate, and mechanically bent to focus the beam at the sample position.

The beamline is operated in two different modes: low-energy mode (LEM) and high-energy mode (HEM). Low-energy mode uses the first harmonics of the undulator beam and the available energy is 6.5–17.5 keV which covers absorption edges of the major anomalous nucleus used in MX. The glancing angle of the mirrors is adjusted to 3.7 mrad, and the focused beam size at the sample position is 80 µm × 22 µm (FWHM, H × V) with a photon flux of 1.1 × 10^13^ photons s^−1^. High-energy mode uses the third harmonics of the undulator beam and covers 20.6–35.4 keV. The mirror glancing angle is 1.5 mrad.

The specifications of the beamline are summarized in Table 1 ▶.

## 3. Beam collimation and experimental environment

### 3.1. Beam collimation with pinhole aperture

**Figure 2:**
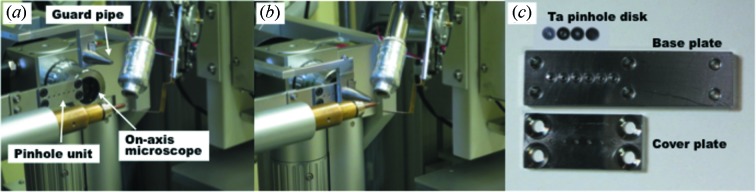
(*a*) Pinhole unit and guard pipe equipped on the diffractometer. They are evacuated during sample mounting and sample centering. (*b*) The pinhole and the guard pipe are inserted into the beam path. (*c*) Pinhole unit disassembled into the pinhole disks, the base plate and the cover plate.

In order to minimize the beam size to 10 µm, we used a pinhole collimator, which is similar to the concept developed at GM/CA-CAT beamlines 23ID-D and 23ID-B at the Advanced Photon Source (Fischetti *et al.*, 2009 ▶). Fig. 2 ▶ shows the pinhole collimation system developed at BL41XU. It is composed of a pinhole unit for beam shaping and a guard pipe to eliminate parasitic scattering from the pinhole [Figs. 2(*a*) and 2(*b*) ▶]. The pinhole unit consists of pinhole disks, a base plate and a cover plate (Fig. 2*c* ▶). The pinhole disk is made of tantalum and has a pinhole at the center. The disks are mounted on the base plate and sandwiched with the cover plate. A maximum of six pinhole disks can be mounted in the pinhole unit. We use four pinholes: 10, 20, 30 and 50 µm in diameter, so that beam size can be changed depending on the sample size. The forward scattering from the pinhole is eliminated by the guard pipe. Its shape is a hollow cone with a height of 30 mm, and a tantalum pinhole with a diameter of 0.1 mm is attached at its tip. The distance between the tip of the guard pipe and the sample is 6 mm.

**Figure 3:**
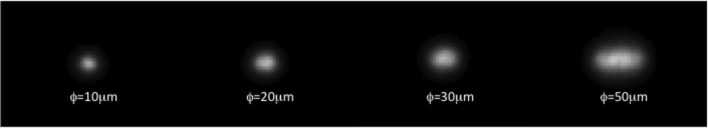
Beam shape observed by a beam monitor placed at the sample position. The beam monitor is composed of a phosphor screen of 10 µm Gd_2_O_2_S:Tb, optical lens and a CCD camera. The pixel size of the image is 3 µm. The beam size is 15.1 µm × 13.6 µm, 21.2 µm × 17.3 µm, 24.9 µm × 18.4 µm and 44.5 µm × 21.4 µm (FWHM, H×V), respectively.

**Figure 4:**
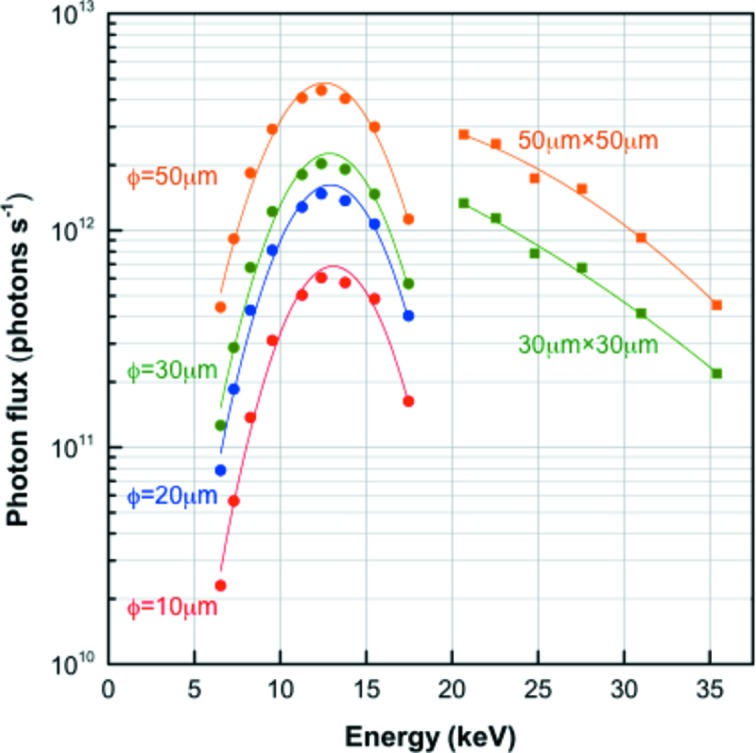
Photon flux *versus* X-ray energy. The four-blade slits were used in HEM instead of pinholes because the high-energy X-rays penetrate the pinhole disks. The HEM data (20.6–35.4 keV) were obtained before the implementation of liquid-nitrogen cooling to the DCM, *i.e* a water-cooling system had been used.

The beam shape obtained by the pinhole system at 12.4 keV is show in Fig. 3 ▶. The beam size of the 10 and 20 µm pinhole beam has a circular shape, whereas the 30 µm and 50 µm pinhole beam has an elliptical shape, which reflects the shape of the beam focused by the mirrors. The energy dependency of the photon flux is plotted in Fig. 4 ▶.

### 3.2. Experimental environment

**Figure 5:**
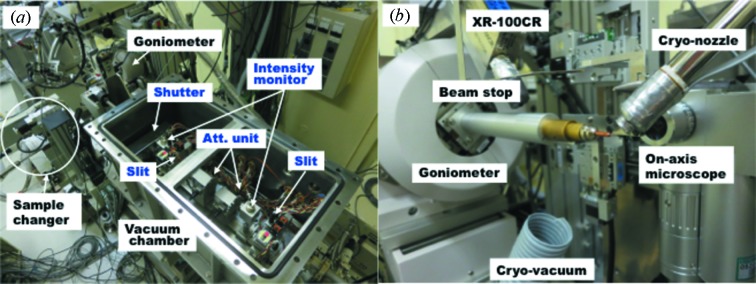
Experimental environment of BL41XU (*a*) inside the vacuum chamber located upstream of the diffractometer. (*b*) Magnified view around the sample position.

Fig. 5 ▶ shows the diffractometer at BL41XU. In order to align and shape the beam, two four-blade slits, a shutter, two intensity monitors and two attenuator units are installed in a vacuum chamber located upstream of the diffractometer (Fig. 5*a* ▶). The intensity monitor consists of a Si PIN photodiode and beryllium foil inserted in the beam path with a tilt of 45°, and measures the X-ray scattering by the foil. The slit was mainly used for the positional tuning of the diffractometer stage by setting the aperture size of 0.03 mm × 0.5 mm. During the data collection the aperture is set to 0.25 mm × 0.25 mm and 0.2 mm × 0.2 mm, respectively, and used only to eliminate the parasitic scattering from the mirror. The attenuator unit consists of a series of aluminium foils attached on the motorized disk. The combination of the two units reduces the intensity to 1/10 to 1/10000 over the available energy range.

The sample environment, shown in Fig. 5(*b*) ▶, is set up to collect diffraction data even from micro-crystals. To visualize the sample, an on-axis microscope with zoom lens (Union Optical, Japan) is installed. Just downstream of the microscope is the pinhole collimation system described in the previous section. The air-bearing goniometer of QSU-0 (Kohzu, Japan) performs a maximum rotation speed of 180° s^−1^ with a low eccentricity of less than 1 µm. The sample is cooled by the cryo-cooler CRYOCOOL-G2b-LT2 (Cryo Industries, USA), which can select either nitrogen or helium as a cooling gas. When the helium gas is used, the sample can be cooled down to 20 K. The sample changer SPACE developed at SPring-8 (Murakami *et al.*, 2012 ▶) is used for automatic sample exchange.

To collect diffraction images, the high-sensitivity CCD detector MX225HE (Rayonix, USA) is used. The minimum camera distance of 70 mm enables us to collect data up to 1 Å at 12.4 keV. The detector position can be vertically offset in order for high-resolution data collection of large-unit-cell crystals. Another option for the detector selection is the imaging plate (IP) detector RAXIS V (Rigaku, Japan). The wide dynamic range of the IP allows collection of the ultra-low-resolution reflections of large-unit-cell crystals. For the fluorescence scan, the Si PIN photodiode detector XR-100CR (AmpTek, USA) is installed.

Data collection is conducted using *BSS*, the standard control software at SPring-8 MX beamlines (Ueno *et al.*, 2005 ▶). It provides tools for advanced data collections such as helical scanning (Flot *et al.*, 2010 ▶) and grid scans (Bowler *et al.*, 2010 ▶; Aishima *et al.*, 2010 ▶), which are now inevitable tools for data collection from difficult targets.

## 4. High-energy mode

One characteristic feature of BL41XU is the capability of data collection in HEM, which provides X-rays of 20.6–35.4 keV with the photon flux shown in Fig. 4 ▶. Since the mirror glancing angle is changed from 3.7 mrad to 1.5 mrad in HEM, the beam position changes by 32 mm in the horizontal direction and by 29 mm in the vertical direction at the sample. Therefore, beamline alignment is needed to switch the operation mode. In HEM, resizing the beam is not conducted by pinhole but by the four-blade slits in the vacuum chamber (Fig. 5*a* ▶), because the high-energy X-rays penetrate the pinhole disks.

The major aim of HEM is ultra-high-resolution data collection. So far, Takeda and colleagues have demonstrated data collection up to 0.5 Å using 31 keV X-rays at BL41XU (Takeda *et al.*, 2010 ▶). Another use of HEM is structure determination using the *K*-absorption edges of atoms such as iodine (33.17 keV) and xenon (34.56 keV) for structure determination (Takeda *et al.*, 2004 ▶). However, the low sensitivity of most X-ray detectors to high-energy photons diminishes the value of this mode. To deal with this problem, we have been developing a CMOS detector which uses CsI as a phosphor material (Hasegawa *et al.*, 2009 ▶).

## 5. Upgrade project

The targets for the structural study are still becoming more challenging and crystals brought to the beamline are becoming smaller. Moreover, need for beam time is increasing. Therefore, we are proceeding with an upgrade project.

In this project we set our goal to achieve variable beam size between 5 µm and 50 µm while keeping the photon flux of the order of 10^13^ photons s^−1^. Fig. 1(*b*) ▶ shows the design of the new beamline optics. The main feature is making use of two-step focusing using figured elliptical mirrors. The beam from the light source is focused at the slit in EH1 by the first horizontal mirror, and then it is imaged to the sample position by the second set of mirrors in KB configuration. Ray-tracing using *SHADOW* (Sánchez del Rio & Dejus, 2004 ▶) showed that the minimum beam size is 5 µm (H) × 10 µm (V) with a photon flux of 1.1 × 10^13^ photon s^−1^ at 12.4 keV. The maximum beam size is expected to be 50 µm (H) × 50 µm (V) and its photon flux will be 5.4 × 10^13^ photon s^−1^. The beam size is changed by combining the following three ways: (i) change of virtual source size, (ii) offset of the sample from the focus position and (iii) change of vertical mirror angle.

Together with the upgrade of the optics, we will replace a diffractometer and a detector. The newly installed detector is a pixel detector with large sensitive area and provides shutterless data collection (Brönnimann *et al.*, 2003 ▶; Ito *et al.*, 2007 ▶).

We suppose that HEM will still be an important feature of BL41XU after the upgrade. In order to set up HEM without deteriorating the specification of LEM and, moreover, without a large modification of the LEM set-up, we will use EH1 for HEM. For this purpose, the second diffractometer will be installed in EH1 (Fig. 1*b* ▶). We plan to use a compound refractive lens as the focusing optics (Snigirev *et al.*, 1996 ▶) for HEM.

## 6. Summary and conclusion

We have developed BL41XU to deal with difficult data collection from small and/or poorly diffracting crystals. The implementation of the pinhole collimation provided a minimum beam size of 10 µm and allows data collection from small crystals down to 10 µm. The pinhole system also provides variable beam size without time-consuming realignment of the optics. The HEM of BL41XU provides the unique opportunity of ultra-high-resolution data collection and use of the *K*-absorption edges of Xe and I. The upgrade of the beamline optics will allow us to use a minimum 5 µm (H) × 10 µm (V) beam with a photon flux of 1.2 × 10^13^ photon s^−1^. Together with the installation of the pixel detector, BL41XU will enable more accurate and more rapid data collection from challenging targets. We therefore believe that BL41XU will continuously contribute to structural biology research.

## Figures and Tables

**Table 1:** Beamline details

Beamline name	BL41XU
Source type	In-vacuum undulator
Monochromator	Double-crystal Si(111) liquid-nitrogen-cooled
Energy range	6.5–17.5 keV (LEM), 20.6–35.4 keV (HEM)
Wavelength range	0.71–1.9 Å (LEM), 0.35–0.6 Å (HEM)
Mirrors	Rh-coated Si, 700 mm (H), 400 mm (V)
Beam size without pinhole (FWHM, H × V)	80 µm × 22 µm (12.4 keV)
Photon flux without pinhole	1.1 × 10^13^ photons s^−1^ (12.4 keV)
Goniometer	QKSU0 (KOHZU)
Cryo capability	CRYOCOOL-G2b-LT2 (Cryo Industries)
Sample mounting	SPACE
CCD detector	MX225HE (Rayonix)
Imaging-plate detector	RAXIS V (Rigaku)
